# Use of cangrelor in patients with acute coronary syndromes undergoing percutaneous coronary intervention: Study design and interim analysis of the ARCANGELO study

**DOI:** 10.1002/clc.23878

**Published:** 2022-06-22

**Authors:** Leonardo De Luca, Paolo Calabrò, Fabio Chirillo, Cristina Rolfo, Alberto Menozzi, Piera Capranzano, Maurizio Menichelli, Elisa Nicolini, Ciro Mauro, Carlo Trani, Francesco Versaci, Fabrizio Tomai, Giuseppe Musumeci, Carlo Di Mario, Martino Pepe, Sergio Berti, Carlo Cernetti, Plinio Cirillo, Diego Maffeo, Giuseppe Talanas, Marco Ferlini, Marco Contarini, Valerio Lanzilotti, Marino Scherillo, Giuseppe Tarantini, Simone Muraglia, Roberta Rossini, Leonardo Bolognese, ARCANGELO study group

**Affiliations:** ^1^ Department of Cardiosciences, Division of Cardiology Azienda Ospedaliera San Camillo‐Forlanini Rome Italy; ^2^ U.O.C. Cardiologia Clinica con UTIC. A.O.R.N. Sant'Anna e San Sebastiano Caserta Italy; ^3^ U.O.C. Cardiologia Ospedale San Bassiano Bassano del Grappa (VI) Italy; ^4^ S.C. Cardiologia Ospedale degli Infermi di Rivoli ASLTO3 Rivoli Italy; ^5^ S.C. Cardiologia, Ospedale S. Andrea, ASL5 Liguria La Spezia Italy; ^6^ U.O. Cardiologia A.O.U. Policlinico G. Rodolico Catania Italy; ^7^ Cardiologia Ospedale Fabrizio Spaziani Frosinone Italy; ^8^ U.O. Cardiologia Interventistica strutturale e pediatrica, Ospedali Riuniti Ancona Italy; ^9^ Cardiologia UTIC con emodinamica AORN Cardarelli Napolii Napoli Italy; ^10^ U.O.C. Interventistica Cardiologica e diagnostica invasiva Fondazione Policlinico Universitario A. Gemelli, IRCCS Rome Italy; ^11^ Department of Cardiology Santa Maria Goretti Hospital Latina Italy; ^12^ U.O.C. di Cardiologia Aurelia Hospital Rome Italy; ^13^ S.C. Cardiologia, A.O. Ordine Mauriziano Torino Italy; ^14^ Interventistica Cardiologica Strutturale A.O.U. Careggi Firenze Italy; ^15^ Cardiologia Universitaria A.O.U. Consorziale Policlinico Bari Italy; ^16^ Fondazione C.N.R. Reg. Toscana G. Monasterio Pisa Italy; ^17^ Cardiologia Ospedale Ca' Foncello Treviso Italy; ^18^ Dipartimento di Scienze Biomediche Avanzate Cardiologia, A.O.U.P. “Federico II” Napoli Italy; ^19^ Cardiologia Emodinamica Fondazione Poliambulanza Brescia Italy; ^20^ U.O.C. Cardiologia Clinica ed Interventistica Azienda Ospedaliero‐Universitaria di Sassari Sassari Italy; ^21^ U.O.C. Cardiologia Fondazione IRCCS Policlinico San Matteo San Matteo Italy; ^22^ U.O.C. di Cardiologia con UTIC ed Emodinamica Ospedale Umberto I di Siracusa Azienda Sanitaria Provinciale di Siracusa Italy; ^23^ U.O.C. Cardiologia Ospedale Maggiore Bologna Italy; ^24^ U.O.C. Cardiologia interventistica e UTIC Azienda Ospedaliera San Pio Benevento Italy; ^25^ U.O.S.D. Emodinamica e Cardiologia Interventistica Azienda Ospedale Università Padova Italy; ^26^ U.O. Cardiologia, Ospedale S. Chiara Trento Italy; ^27^ Cardiologia ASO Santa Croce e Carle Cuneo Italy; ^28^ Cardiologia Ospedale San Donato Arezzo Italy

**Keywords:** acute coronary syndrome, bleeding, cangrelor, cardiac artery disease, P2Y12 inhibitor, real‐world evidence

## Abstract

**Background:**

The itAlian pRospective Study on CANGrELOr (ARCANGELO) was aimed to assess the safety of using cangrelor during percutaneous coronary intervention (PCI) in patients with acute coronary syndromes (ACS) in the daily practice.

**Hypothesis:**

The safety of cangrelor after the transition to oral P2Y12 inhibitors was evaluated as the incidence of bleeding outcomes in the 30 days following PCI according to postauthorization safety study guidelines.

**Methods:**

Adults with ACS who were treated with cangrelor in one of the 28 centers involved in the study. Patients who consented to participate were followed in the 30 days following their PCI. Bleedings (Bleeding Academic Research Consortium [BARC] classification), major adverse cardiac events (MACEs), and adverse events were recorded. The interim results at two‐thirds of the enrollment period are presented.

**Results:**

A total of 17 bleedings were observed in the 320 patients who completed the study at this stage. All bleedings were classified as BARC Type 1–2, except for one case of Type 3a (vessel puncture site hematoma). Four patients experienced MACEs (2 acute myocardial infarctions, 1 sudden cardiac death, 1 noncardiovascular death due to respiratory distress, and multiorgan failure). None of the bleedings was rated as related to cangrelor.

**Conclusions:**

The interim results of the ARCANGELO study provide a preliminary confirmation that the use of cangrelor on patients with ACS undergoing PCI is not associated with severe bleedings.

## 1 INTRODUCTION

Acute coronary syndromes (ACS) are widely and successfully treated with percutaneous coronary intervention (PCI) with stent implantation.^1^, ^2^, ^3^, ^4^, ^5^ Despite their undoubted effectiveness, oral P2Y12 receptor inhibitors^4^, ^5^, ^6^, ^7^ have several limitations when they are used for the urgent or periprocedural treatment of patients with cardiovascular disease who may undergo PCI, including a delayed onset of action.^8^ These limitations are critical in patients in the acute phase of cardiovascular illness, who can be sedated, intubated, in shock, or have nausea, impaired absorption, or impaired perfusion that cannot allow drug administration, limiting oral P2Y12 inhibitors bioavailability.^9^, ^10^, ^11^, ^12^, ^13^ Nausea and vomiting have been reported in almost two‐thirds of patients with ST‐segment elevation myocardial infarction (STEMI).^14^ These limitations can be particularly problematic in the acute care setting surrounding PCI, making thrombotic complications during PCI a major concern.^7^, ^15^

Cangrelor is the only intravenous P2Y12 inhibitor available that can avoid these deficiencies by achieving fast and strong platelet inhibition in all clinical scenarios.^16^, ^17^ Extensive platelet inhibition is maintained throughout the infusion period with the near‐full recovery of platelet function within 60–90 minutes of terminating the infusion.^18^

Trials that led to the cangrelor approval were mainly performed on patients with non‐ST‐segment elevation myocardial infarction (NSTEMI) and unstable angina (UA),^19^, ^20^, ^21^ but cangrelor has increasingly been used in STEMI in real‐life practice.^22^, ^23^, ^24^, ^25^

European Medicines Agency (EMA) suggested to the marketing authorization holder of cangrelor (Chiesi) to perform a postauthorization nonimposed safety study (Category 3)^26^ focused on ACS, collecting information from the daily clinical practice, to evaluate the safety of the transition from cangrelor to any oral P2Y12 inhibitor on the marketed product.^27^ The design, rationale, and preliminary results from the interim analysis of the itAlian pRospective Study on CANGrELOr (ARCANGELO) will be presented in this article.

## 2 METHODS

### 2.1 The ARCANGELO study

The ARCANGELO study aimed to assess the safety of cangrelor in a real‐world setting when it is administered in patients with ACS undergoing PCI who had not received an oral P2Y12 inhibitor before the PCI procedure and in whom oral therapy with P2Y12 inhibitors was not feasible or desirable. The safety of cangrelor was evaluated as the incidence of bleeding outcomes in the 30 days post‐PCI. Secondary endpoints of the study included the assessment of the efficacy of cangrelor in terms of incidence of major adverse cardiac events (MACEs), at 48 hours and 30 days post‐PCI, including death, myocardial infarction (MI), ischemia‐driven revascularization and stent thrombosis (ST). Furthermore, the safety related to the management of transitions from cangrelor to each oral platelet P2Y12 inhibitor (prasugrel/ticagrelor/clopidogrel) was investigated.^27^, ^28^

This observational, prospective cohort study included patients who received cangrelor intravenous transitioning to oral clopidogrel, prasugrel, or ticagrelor in a real‐world setting^29^ between October 23, 2020 and December 1, 2021.

Because of the exploratory nature of the current study, no formal hypotheses were prespecified. The sample size was defined according to feasibility considerations with respect to the duration of the enrollment period and the annual volume of patients managed by the selected sites that were involved in the study. It was estimated that 1000 patients could be enrolled in approximately 12 months, considering the PCI volume of the participating centers. Less than 10% of the enrolled patients were expected not to be evaluable for the primary analysis (i.e., due to violations of eligibility criteria or missing information on primary outcomes).

Therefore, 900 patients were expected to be available for the evaluation of the study endpoint. Simulations were performed to estimate the achievable precision of the 95% confidence interval (95% CI) of the expected proportions, assuming 900 evaluable patients. Expected proportions were defined according to the available literature showing a relative error ranging from 14.2% (any noncoronary artery bypass grafting‐related Global Use of Strategies to Open Occluded Arteries [GUSTO] bleeding, at an expected frequency of 17.5%) to 49.7%.^28^

**Figure 1:**
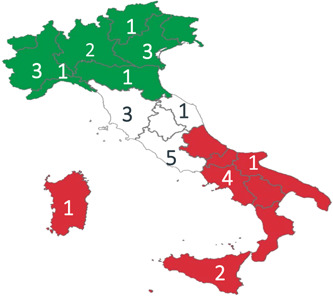
Location of the 28 Italian study centers participating in the itAlian pRospective Study on CANGrELOr study

Adults (>18 years) undergoing PCI for ACS and treated with cangrelor in one of the 28 centers involved in the study (Figure 1) were eligible to be included if providing both their informed and privacy consent within the observational period.^28^

The ARCANGELO study was registered before the beginning of patient enrollment (registration number NCT04471870).

The study evaluated the incidence of any hemorrhages, calculated as the ratio between the number of patients experiencing at least one event during the 30‐day observation period over the total number of evaluable patients, according to Bleeding Academic Research Consortium (BARC) criteria.^30^ The different types of bleedings at various timeframes (from 48 hours to 30 days) according to the GUSTO criteria^31^ and MACEs at various timeframes (from 48 hours to 30 days) were investigated, too.

The proportion of patients receiving any of the oral platelet P2Y12 receptor inhibitor agents was evaluated as type and timing of administration. Adverse events and reactions, including adverse drug reactions (ADRs), were collected and their relationship with the therapies was rated by each investigator.

A continuous, detailed, predetermined monitoring of the study was performed at the start of the study, regularly throughout the study, and after study completion. During monitoring visits, all study records including electronic case report form (eCRF), investigator study file, and source data, were checked ensuring patients' confidentiality. Furthermore, the compliance with the study protocol was verified and any emergent problem was discussed before the validation of the data collected in the eCRFs both for accuracy and completeness against the source documents.

The results of the statistical analyses were summarized by descriptive statistics including frequency, count, and percentage for categorical variables, the number of observations, mean ± standard deviation (SD), median, 25th percentile, 75th percentile, minimum, and maximum for continuous variables.

## 3 RESULTS

**Figure 2:**
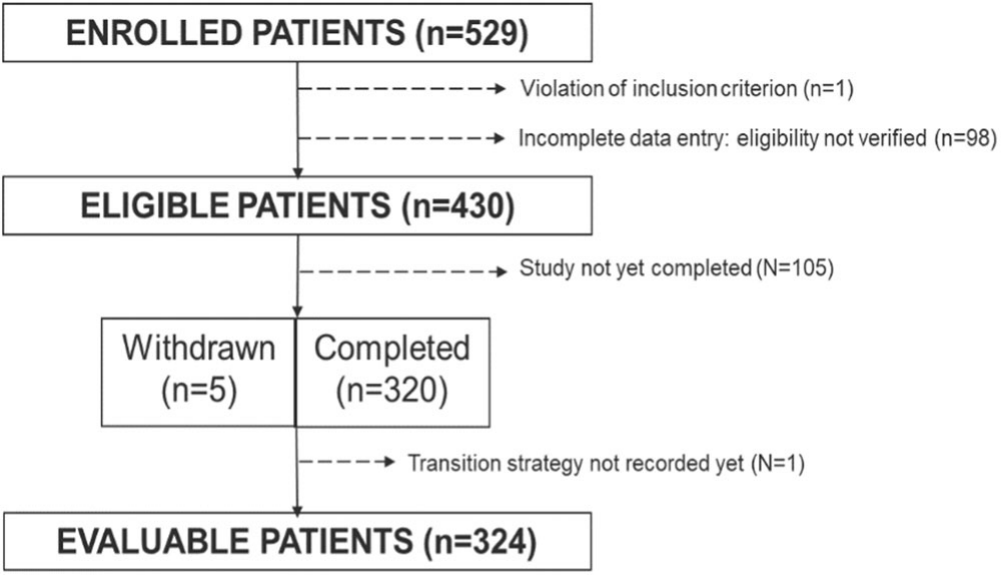
Patient disposition of the itAlian pRospective Study on CANGrELOr study

At two‐thirds of the enrollment period (July 1, 2021), 529 patients were enrolled in the study; 320 of them had completed the 30‐day observation period after the PCI. One of them was excluded from this analysis because complete data were missing at the time of the database extraction. Five patients were prematurely withdrawn, due to loss to follow‐up (*n* = 2), death (*n* = 2), and withdrawal of consent (*n* = 1). Evaluable patients included in this analysis were, thus, 324 (Figure 2).

The mean ± SD duration of observation from PCI to the final study visit was 31.1 ± 4.0 days (median: 31 days; 25th–75th percentiles: 30–32 days). The patient's mean and median age was 65.0 years; 240 (74.1%) were males; 250 (77.2%) had at least one comorbidity, hypertension being the most frequent of them (195 [60.2%] of the patients). A total of 204 (63.0%) patients had STEMI, as cardiovascular disease, 86 (26.5%) NSTEMI, and 34 (10.5%) UA. A total of 164 (50.6%) patients had a single vessel coronary artery disease (Table 1). Three hundred and one (92.9%) of the reference PCIs were performed using radial access, with the implant of a drug‐eluting stent in 98.5% of the cases (Table 2).

**Table 1:** Demographic and clinical characteristics

	Evaluable patients, *N* = 324
Age at enrollment (years)	
*N*	324
Mean ± SD	65.0 ± 11.0
Median (25th–75th percentiles)	65 (57–73)
Minimum; maximum	29; 91
Age at enrollment (classes)	
<75 years	256 (79.0%)
≥75 years	68 (21.0%)
Gender, *n* (%)	
Male	240 (74.1%)
Female	84 (25.9%)
Type of ACS, *n* (%)	
STEMI	204 (63.0%)
NSTE‐ACS	120 (37.0%)
NSTEMI	86 (26.5%)
UA	34 (10.5%)
Type of CAD, *n* (%)	
Monovessel	164 (50.6%)
Multivessel	160 (49.4%)
Two vessels	98 (30.2%)
Three vessels	48 (14.8%)
Detail of CAD, *n* (%)^a^	
Proximal LAD coronary artery	199 (61.4%)
Left circumflex artery	112 (34.6%)
Right coronary artery	160 (49.4%)
Left main disease	20 (6.2%)
Other(s)	61 (18.8%)
Comorbidities, *n* (%)^a^	
Any	250 (77.2%)
Hypertension	195 (60.2%)
Hyperlipidemia	137 (42.3%)
Diabetes	61 (18.8%)
Obesity	18 (5.6%)
Hypothyroidism	15 (4.6%)
Peripheral‐artery disease	15 (4.6%)
COPD	11 (3.4%)
CKD	7 (2.2%)
Other	65 (26.0%)

Abbreviations: ACS, acute coronary syndrome; CAD, coronary artery disease; CKD, chronic kidney disease; COPD, chronic obstructive pulmonary disease; LAD, left anterior descending; NSTE‐ACS, non‐ST‐elevation acute coronary syndromes; NSTEMI, non‐ST‐segment elevation myocardial infarction; STEMI, ST‐segment elevation myocardial infarction; UA, unstable angina.

^a^ The same patient could have more than one option for: “Detail of coronaropathy” or “comorbidities.”

**Table 2:** Details of PCI

	Evaluable patients, *N* = 324
Catheter access site(s), *n* (%)^a^	
Radial	301 (92.9%)
Femoral	29 (9.0%)
Brachial	1 (0.3%)
Type of implanted stent, *n* (%)^a^	
DES	319 (98.5%)
Patients distribution by no. of vessels with DES implantation	
One vessel	261 (80.6%)
Two vessel	52 (16.0%)
Three vessels	6 (1.9%)

Abbreviations: DES, drug‐eluting stent; PCI, percutaneous coronary intervention.

^a^ The same patient could have more than one option for: “Catheter access site(s)” and “type of implanted stent.”

The mean ± SD total duration of cangrelor infusion accounted for 149.9 ± 47.2 minutes; total infusion duration was a maximum of 4 hours for 303 patients (93.5%), while 14 patients (4.3%) received cangrelor for >4 hours (but not more than 6 hours as for site normal clinical practice); in 7 cases (2.2%), duration of cangrelor was not available.

In one patient the duration of cangrelor administration lasted less than 2 hours without the occurrence of any ADRs.

A total of 230 patients (71.0%) received ticagrelor, 50 (15.4%) received prasugrel, and 44 (13.6%) received clopidogrel as oral P2Y12 inhibitor treatment as a transition strategy. The 98 patients who transitioned to ticagrelor took the oral P2Y12 inhibitor after a median of 0 minutes (25th–75th percentiles: 0–10; min: 0; max: 270) after stopping the infusion of cangrelor; the 95 patients who transitioned to ticagrelor before the end of the cangrelor infusion were administered the oral drug 30 minutes (25th–75th percentiles: 30–47; min: 5; max: 279) before the end of the intravenous P2Y12 inhibitor administration. The 31 patients who transitioned to prasugrel took it after a median of 0 minutes (25th–75th percentiles: 0–1; min: 0; max: 35) after stopping the cangrelor infusion; the 17 patients who transitioned to prasugrel before the end of the cangrelor infusion took the oral P2Y12 inhibitor 30 minutes (25th–75th percentiles: 30–30; min: 3; max: 54) before the end of the intravenous P2Y12 inhibitor. The 34 patients who transitioned to clopidogrel took the drug after a median of 0 minutes (25th–75th percentiles: 0–15; min: 0; max: 60) after stopping the cangrelor infusion; the four patients who transitioned to clopidogrel before the end of the intravenous P2Y12 inhibitor infusion took the oral drug 15 minutes (25th–75th percentiles: 13–23; min: 11; max: 30) before the end of the cangrelor administration. The transition exact timings were not available in the eCRF, during data extraction, for the other patients.

Sixteen patients of this cohort experienced at least one bleeding event during the observation period. Only 1 patient experienced 2 bleeding events; therefore, a total of 17 bleedings were observed, 5 of which occurred within the 48 hours following the intervention. All bleedings were classified as BARC Type 1–2, except for one case of Type 3a (vessel puncture site hematoma). The most frequent types were ecchymosis (*n* = 4), bleedings in the urinary tract (i.e., hematuria and urethral hemorrhage; *n* = 4), and hematomas at vascular access site/vessel puncture site (*n* = 4). The study investigators did not rate any of the bleeding events as probably, possibly, or certainly related to cangrelor.

A total of 11 bleedings were rated as related to PCI, 9 as related to other drugs (6 probably/possibly related to oral P2Y12 receptor inhibitors) (Table 3).

**Table 3:** Detailed description of the observed bleeding events

Bleeding #	Bleeding type	Bleeding severity (BARC criteria)	Bleeding severity (GUSTO criteria)	Bleeding correlation to PCI^a^	Bleeding correlation to concomitant drugs
1	Epistaxis	Type 1	Mild	No	Probable (ASA, ticagrelor)
2	Lower Gi hemorrhage	Type 2	Moderate	No	Probable (prasugrel/ASA)
3	Hematuria	Type 1	Mild	Probable	Probable (ASA, ticagrelor)
4	Arterial bleeding	Type 2	Mild	Possible	No
5	Hematuria	Type 2	Mild	No	Probable (prasugrel)
6	Epistaxis	Type 1	Mild	No	Probable (clopidogrel)
7^b^	Catheter site hematoma	Type 1	Mild	Possible	Probable (eptifibatide)
8^b^	Catheter site hematoma	Type 2	Moderate	Certain	No
9	Hematuria	Type 1	Mild	Possible	Possible (UFH)
10	Vessel puncture site hematoma	Type 2	Mild	Certain	No
11	Subcutaneous bleeding	Type 1	Mild	No	No
12	Vessel puncture site hematoma	Type 3a	Moderate	Certain	Probable (UFH)
13	Ecchymosis	Type 1	Mild	Certain	No
14	Ecchymosis	Type 1	Mild	Certain	No
15	Ecchymosis	Type 1	Mild	Certain	No
16	Ecchymosis	Type 1	Mild	Certain	No
17	Urethral hemorrhage	Type 1	Mild	No	Possible (ticagrelor)

Abbreviations: ASA, acetylsalicylic acid; BARC, Bleeding Academic Research Consortium; GI, gastrointestinal; GUSTO, Global Use of Strategies to Open Occluded Arteries; PCI, percutaneous coronary intervention; UFH, unfractionated heparin.

^a^ The relationship between bleeding events and PCI was rated by the study investigator.

^b^ The same patient experienced these bleeding events.

No ADRs related to cangrelor were reported.

Cangrelor was used off‐label in nine patients: Five patients received an oral platelet P2Y12 receptor antagonist (ticagrelor) 24 hours before cangrelor administration, while two patients received ticagrelor or prasugrel more than 30 minutes before cangrelor discontinuation and one patient received clopidogrel before cangrelor discontinuation. Four patients experienced MACEs during the observational period: two patients experienced acute MI, one sudden cardiac death, and one noncardiovascular death due to respiratory distress and multiorgan failure.

A total of 298 monitoring visits were performed during the study.

## 4 DISCUSSION

Most real‐world evidence on the use of cangrelor is derived from retrospective analyses.^23^, ^32^, ^33^, ^34^ These assessments were performed by extracting the data from available clinical databases that may lack the systematic collection of safety data, and thus their outcomes could be based on a limited and not‐systematically collected and rigorously controlled set of data. Furthermore, registration trials of cangrelor were performed only comparing this intravenous P2Y12 inhibitor with the oral P2Y12 inhibitor clopidogrel, which was also the only oral drug used in the transition phase.^35^ In real‐world practice, the more commonly used oral P2Y12 inhibitor transition therapy is ticagrelor,^34^ underscoring the need for real‐world prospective evaluations providing insights on the safety and efficacy of cangrelor in daily clinical use.

The outcomes of this interim analysis, performed on approximately one‐third of the target sample of the ARCANGELO study, confirm the safety of using cangrelor during PCIs. Only one moderate BARC 3a bleeding was observed, no severe bleedings occurred, and no adverse reactions to cangrelor have been reported.

The most recent analysis of the use of antiplatelet therapy in Italian coronary care units was performed in March 2014 when cangrelor was not yet available. In this study aspirin, bivalirudin, and glycoprotein IIb/IIIa inhibitors (GPIs) were more frequently administered treatments before or during PCI. Crossover of heparin therapy occurred in 36.0% of cases, whereas switching from one P2Y12 inhibitor to another occurred in 3.7% of the patients. Furthermore, the multivariable analysis yielded several independent predictors of GPIs and bivalirudin use in the catheterization laboratory, mainly related to clinical presentation, PCI complexity, and the presence of complications during the procedure.^24^, ^36^, ^37^ The preliminary results of the ARCANGELO study show a deep change in the PCI procedures in the Italian hemodynamic centers and that the use of cangrelor seems to contribute to a more standardized and clinically effective approach. Concerning exposure, all 324 patients received cangrelor according to dose and regimen (bolus plus infusion) in the European Union‐Summary of Product Characteristics (EU‐SmPC), with a maximum infusion length of 4 hours for 303 patients (93.5%) and no patients receiving cangrelor for more than 6 hours. In terms of transition strategy from cangrelor to oral P2Y12 receptor inhibitors, ticagrelor was the most used drug (71.0%), followed by prasugrel and clopidogrel (15.4% and 13.6%, respectively); most of the patients were transitioned according to the SmPC, except the few patients who received ticagrelor or prasugrel more than 30 minutes before cangrelor discontinuation or before the end of cangrelor administration. In a recent study on PCI, BARC‐defined bleeding Type 3 or 5 occurred in 0.8%–1.5% of the patients who received ticagrelor or clopidogrel plus aspirin.^38^ The preliminary results from the ARCANGELO study show that BARC Grade 3a bleeding occurred in 1 (0.3%) patient while more severe bleedings were not reported. Even if, differently in other studies on cangrelor,^24^, ^39^, ^40^ 204 (63%) of the patients included in the ARCANGELO study had STEMI, there were no differences in the frequency of bleeding when comparing the different subpopulations.

In this preliminary analysis of the ARCANGELO study, the observed rate of MACE was 1.2% in the 30 days following the PCI. The relevance of these results will be evaluated on the whole population of the ARCANGELO study.

These are preliminary analyses from an observational trial, and thus any clinical outcome must be considered preliminary, needing to be confirmed in a rigorously controlled trial.

The ARCANGELO study was designed according to regulatory authorities' stringent requirements and conducted ensuring levels of quality that are set as of today gold standard, to ensure the reliability of the collected data and the quality of the outcomes.

## 5 CONCLUSION

The design and the interim results of the ARCANGELO study provide a preliminary confirmation that the use of cangrelor in patients with ACS undergoing PCI, following the product's specifications of use, is not associated with severe bleedings, and the benefit–risk balance of cangrelor remains favorable.

The final analysis of the full patient sample will allow a more complete and precise evaluation of the study endpoints.

## ARCANGELO Study Group

Paolo Calabrò (U.O.C. Cardiologia Clinica con UTIC. A.O.R.N. Sant'Anna e San Sebastiano, Caserta); Fabio Chirillo (U.O.C. Cardiologia, Ospedale San Bassiano, Bassano del Grappa [VI]); Cristina Rolfo (S.C. Cardiologia Ospedale degli Infermi di Rivoli ASLTO); Alberto Menozzi (S.C. Cardiologia, Ospedale S. Andrea, La Spezia, ASL5 Liguria); Piera Capranzano (U.O. Cardiologia A.O.U. Policlinico G. Rodolico, Catania); Maurizio Menichelli (Cardiologia Ospedale Fabrizio Spaziani, Frosinone); Elisa Nicolini (U.O. Cardiologia Interventistica, strutturale e pediatrica, Ospedali Riuniti Ancona); Ciro Mauro (Cardiologia UTIC con emodinamica A.O.R.N. Cardarelli Napoli); Carlo Trani (U.O.C. Interventistica Cardiologica e diagnostica invasiva, Fondazione Policlinico Universitario A. Gemelli, IRCCS, Rome); Francesco Versaci (Department of Cardiology, Santa Maria Goretti Hospital, Latina); Fabrizio Tomai (U.O.C. di Cardiologia Aurelia Hospital); Giuseppe Musumeci (S.C. Cardiologia, A.O. Ordine Mauriziano, Torino); Leonardo De Luca (Cardiologia UTIC Ospedale San Camillo Forlanini, Rome); Carlo Di Mario (Interventistica Cardiologica Strutturale A.O.U. Careggi, Firenze); Martino Pepe (Cardiologia Universitaria A.O.U. Consorziale Policlinico); Sergio Berti (Fondazione C.N.R. Reg. Toscana G. Monasterio, Pisa); Carlo Cernetti (Cardiologia Ospedale Ca' Foncello, Treviso); Plinio Cirillo (Dipartimento di Scienze Biomediche Avanzate, Cardiologia, A.O.U.P. “Federico II”, Napoli); Diego Maffeo (Cardiologia Emodinamica Fondazione Poliambulanza, Brescia); Talanas (U.O.C. Cardiologia Clinica ed Interventistica Ospedale SS Annunziata, Sassari); Marco Ferlini (U.O.C. Cardiologia Fondazione IRCCS Policlinico San Matteo, Pavia); Marco Contarini (U.O.C. di Cardiologia con UTIC ed Emodinamica Ospedale Umberto I di Siracusa Azienda Sanitaria Provinciale di Siracusa); Valerio Lanzilotti (U.O.C. Cardiologia Ospedale Maggiore, Bologna); Marino Scherillo (U.O.C. Cardiologia interventistica e UTIC Azienda Ospedaliera San Pio, Benevento); Giuseppe Tarantini (U.O.S.D. Emodinamica e Cardiologia Interventistica Azienda Ospedale Università Padova); Simone Muraglia (U.O. Cardiologia, Ospedale S. Chiara, Trento); Roberta Rossini (Cardiologia ASO Santa Croce e Carle, Cuneo); Leonardo Bolognese (Cardiologia Ospedale San Donato, Arezzo).

## CONFLICT OF INTEREST

Dr. De Luca declares that he has received consulting fees or honoraria from Amgen, Aspen, AstraZeneca, Bayer, Boehringer Ingelheim, Chiesi, Daiichi Sankyo, Eli Lilly, Menarini, Pfizer/Bristol‐Myers Squibb, Sanofi, Servier, and The Medicines Company, outside the present work.

## Data Availability

The data that support the findings of this study are available from the corresponding author upon reasonable request.

## References

[1] Bhatt DL , Roe MT , Peterson ED , et al. Utilization of early invasive management strategies for high‐risk patients with non‐ST‐segment elevation acute coronary syndromes: results from the CRUSADE Quality Improvement Initiative. JAMA. 2004;292:2096‐2104. 10.1001/JAMA.292.17.2096 15523070

[2] Bavry AA , Kumbhani DJ , Rassi AN , Bhatt DL , Askari AT . Benefit of early invasive therapy in acute coronary syndromes: a meta‐analysis of contemporary randomized clinical trials. J Am Coll Cardiol. 2006;48:1319‐1325. 10.1016/J.JACC.2006.06.050 17010789

[3] Mehta SR , Cannon CP , Fox KAA , et al. Routine vs selective invasive strategies in patients with acute coronary syndromes: a collaborative meta‐analysis of randomized trials. JAMA. 2005;293:2908‐2917. 10.1001/JAMA.293.23.2908 15956636

[4] Collet JP , Thiele H , Barbato E , et al. 2020 ESC Guidelines for the management of acute coronary syndromes in patients presenting without persistent ST‐segment elevation. Eur Heart J. 2021;42:1289‐1367. 10.1093/EURHEARTJ/EHAA575 32860058

[5] Amsterdam EA , Wenger NK , Brindis RG , et al. 2014 AHA/ACC Guideline for the management of patients with non‐ST‐elevation acute coronary syndromes: a report of the American College of Cardiology/American Heart Association Task Force on Practice Guidelines. J Am Coll Cardiol. 2014;64:e139‐e228. 10.1016/J.JACC.2014.09.017 25260718

[6] Mehra MR , Desai SS , Kuy S , Henry TD , Patel AN . Cardiovascular disease, drug therapy, and mortality in Covid‐19. N Engl J Med. 2020;25:102. 10.1056/nejmoa2007621 PMC7206931 32356626

[7] Desai NR , Bhatt DL . The state of periprocedural antiplatelet therapy after recent trials. JACC Cardiovasc interv. 2010;3:571‐583. 10.1016/J.JCIN.2010.04.008 20630450

[8] Bhatt DL , Stone GW , Mahaffey KW , et al. Effect of platelet inhibition with cangrelor during PCI on ischemic events. N Engl J Med. 2013;368:1303‐1313. 10.1056/NEJMOA1300815 23473369

[9] Heestermans AACM , van Werkum JW , Taubert D , et al. Impaired bioavailability of clopidogrel in patients with a ST‐Segment elevation myocardial infarction. Thromb Res. 2008;122:776‐781. 10.1016/J.THROMRES.2008.01.021 18455217

[10] Součková L , Opatřilová R , Suk P , et al. Impaired bioavailability and antiplatelet effect of high‐dose clopidogrel in patients after cardiopulmonary resuscitation (CPR). Eur J Clin Pharmacol. 2013;69:309‐317. 10.1007/S00228-012-1360-0 22890586

[11] Alexopoulos D , Varlamos C , Mpahara A , Lianos I . P2Y12 inhibitors for the treatment of acute coronary syndrome patients undergoing percutaneous coronary intervention: current understanding and outcomes. Expert Rev Cardiovasc Ther. 2019;17:717‐727. 31583920 10.1080/14779072.2019.1675513

[12] Agrawal K , Bhatt DL . Antiplatelet therapy: does prasugrel or ticagrelor suffice in patients with STEMI? Nat Rev Cardiol. 2013;10:121‐122. 10.1038/NRCARDIO.2012.199 23319096

[13] Basu J , Sharma S . Early recognition vital in acute coronary syndrome. Practitioner. 2016;260:19‐23. 29016088

[14] Leonardi S , Mahaffey KW , White HD , et al. Rationale and design of the cangrelor versus standard therapy to Achieve Optimal Management of Platelet Inhibition PHOENIX Trial. Am Heart J. 2012;163:768‐776.e2. 10.1016/j.ahj.2012.02.018 22607853

[15] De Luca L , Valgimigli M . Unravelling the puzzle of antithrombotic therapies for complex percutaneous coronary intervention. Eur Heart J Cardiovasc Pharmacother. 2021;7:352‐359. 10.1093/EHJCVP/PVAA107 33175148

[16] Ferreiro JL , Ueno M , Angiolillo DJ . Cangrelor: a review on its mechanism of action and clinical development. Expert Rev Cardiovasc Ther. 2009;7:1195‐1201. 19814662 10.1586/erc.09.101

[17] Sible AM , Nawarskas JJ . Angrelor: a new route for P2Y12 inhibition. Cardiol Rev. 2017;25:133‐139. 28379902 10.1097/CRD.0000000000000142

[18] Akers WS , Oh JJ , Oestreich JH , Ferraris S , Wethington M , Steinhubl SR . Pharmacokinetics and pharmacodynamics of a bolus and infusion of cangrelor: a direct, parenteral P2Y12 receptor antagonist. J Clin Pharmacol. 2010;50:27‐35. 10.1177/0091270009344986 19779037

[19] Cavender M , Harrington R , Stone G , et al. Ischemic events occur early in patients undergoing PCI and are reduced with cangrelor: findings from champion Phoenix. J Am Coll Cardiol. 2017;69:25. 10.1016/s0735-1097(17)33414-9 28057246

[20] Bhatt DL , Stone GW , Mahaffey KW , et al. Effect of platelet inhibition with cangrelor during PCI on ischemic events. N Engl J Med. 2013;368:1303‐1313. 10.1056/NEJMoa1300815 23473369

[21] Cavender M , Harrington R , Stone G , et al. Early benefit of cangrelor in patients undergoing PCI in CHAMPION PHOENIX. Eur Heart J. 2014;35:P732.

[22] Vaduganathan M , Qamar A , Badreldin HA , Faxon DP , Bhatt DL . Cangrelor use in cardiogenic shock: a single‐center real‐world experience. JACC Cardiovasc Interv. 2017;10:1712‐1714. 10.1016/j.jcin.2017.07.009 28838482

[23] Grimfjärd P , Lagerqvist B , Erlinge D , Varenhorst C , James S . Clinical use of cangrelor: nationwide experience from the Swedish Coronary Angiography and Angioplasty Registry (SCAAR). Eur Heart J Cardiovasc Pharmacother. 2019;5:151‐157. 10.1093/ehjcvp/pvz002 30698669

[24] De Luca L , Steg PG , Bhatt DL , Capodanno D , Angiolillo DJ . Cangrelor: clinical data, contemporary use, and future perspectives. J Am Heart Assoc. 2021;10:22125. 10.1161/JAHA.121.022125 PMC8403274 34212768

[25] Pepe M , Larosa C , Cirillo P , et al. Clinical use of cangrelor: a real world multicenter experience from South Italy insights from the M.O.Ca. Registry. Panminerva Med. 2021;64:9‐16. 10.23736/S0031-0808.21.04437-2 34060281

[26] European Medicines Agency. Guideline on Good Pharmacovigilance Practices (GVP)—Module VIII—Post‐Authorisation Safety Studies (Rev 3). 2017.

[27] European Medicines Agency. Part VI: Summary of Activities in the Risk Management Plan 2013.

[28] Chiesi Farmaceutici S.p.A. ARCANGELO (ItAlian PRospective Study on CANGrELOr). Accessed November 26, 2020. https://clinicaltrials.gov/ct2/show/NCT04471870?term=arcangelo%26draw=2%26rank=1

[29] ANNEX I. Summary of product characteristics. European Medicines Agency. Accessed November 26, 2020. https://www.ema.europa.eu/en/documents/product-information/kengrexal-epar-product-information_en.pdf

[30] Wells GA , Elliott J , Kelly S , et al. *Bleeding Classification System Definitions*. Canadian Agency for Drugs and Technologies in Health; 2019.

[31] Mehran R , Rao Sv , Bhatt DL , et al. Standardized bleeding definitions for cardiovascular clinical trials. Circulation. 2011;123:2736‐2747. 10.1161/CIRCULATIONAHA.110.009449 21670242

[32] Vaduganathan M , Qamar A , Singh A , et al. Cangrelor use since FDA approval: a single‐center, real‐world experience at a tertiary care hospital. J Am Coll Cardiol. 2017;69:463‐464. 27914699 10.1016/j.jacc.2016.11.017

[33] Rossini R , Masiero G , Fruttero C , et al. Antiplatelet therapy with cangrelor in patients undergoing surgery after coronary stent implantation: a real‐World bridging protocol experience. TH Open. 2020;04:e437‐e445. 10.1055/s-0040-1721504 PMC7758156 33376943

[34] Droppa M , Spahn P , Takhgiriev K , et al. Periprocedural platelet inhibition with cangrelor in P2Y12‐inhibitor naïve patients with acute coronary syndromes—a matched‐control pharmacodynamic comparison in real‐World patients. Int J Cardiol. 2016;223:848‐851. 10.1016/j.ijcard.2016.08.270 27580219

[35] Bhatt DL , Lincoff AM , Gibson CM , et al. Intravenous platelet blockade with cangrelor during PCI. N Engl J Med. 2009;361:2330‐2341. 10.1056/nejmoa0908629 19915222

[36] De Luca L , Musumeci G , Leonardi S , et al. Antithrombotic strategies in the catheterization laboratory for patients with acute coronary syndromes undergoing percutaneous coronary interventions: insights from the EmploYEd antithrombotic therapies in patients with acute coronary syndromes hospitalized in Italian Cardiac Care Units Registry. J Cardiovasc Med. 2017;18:580‐589. 10.2459/JCM.0000000000000533 28639987

[37] de Luca L , Leonardi S , Cavallini C , et al. Contemporary antithrombotic strategies in patients with acute coronary syndrome admitted to cardiac care units in Italy: the EYESHOT study. Eur Heart J Acute Cardiovasc Care. 2015;4:441‐452. 10.1177/2048872614560505 25414322

[38] Tomaniak M , Chichareon P , Onuma Y , et al. Benefit and risks of aspirin in addition to ticagrelor in acute coronary syndromes: a post hoc analysis of the randomized Global Leaders Trial. JAMA Cardiol. 2019;4:1092‐1101. 10.1001/JAMACARDIO.2019.3355 31557763 PMC6764000

[39] Keating GM . Cangrelor: a review in percutaneous coronary intervention. Drugs. 2015;75:1425‐1434. 10.1007/s40265-015-0445-3 26201463

[40] Feng KY , Mahaffey KW . Cangrelor in clinical use. Future Cardiol. 2020;16:89‐102. 32067479 10.2217/fca-2019-0095

